# Defining Multiple Fibroadenomas: A Systematic Review of Clinical Characteristics and Management

**DOI:** 10.1155/tbj/5572826

**Published:** 2026-04-01

**Authors:** Prathiksha Math, Gayathri Sajith, Kiran Mahadevappa, Shalini G. Hegde

**Affiliations:** ^1^ Department of Paediatric Surgery, St. John’s Medical College Hospital, Bangalore, Karnataka, India; ^2^ Department of Cardiology, St. John’s Medical College Hospital, Bangalore, Karnataka, India

**Keywords:** adolescent, breast diseases, breast, fibroadenoma, multiple fibroadenoma

## Abstract

This review systematically evaluates the published literature on multiple fibroadenomas (MFAs) of the breast, focusing on clinical presentation, diagnostic challenges, and treatment strategies. A systematic search of PubMed, Medline, Scopus, and Embase from 1948 to June 2025 was conducted using a Boolean search strategy following the PRISMA 2020 guidelines. Of the 409 studies initially screened, 43 met the inclusion criteria. The level of evidence was assessed using the Oxford Centre for Evidence‐Based Medicine guidelines, and 72% were found to be of Levels 4 and 5. MFA most commonly affects adolescents. Bilateral involvement was observed in 64.6% of patients, and more than 10 lesions were present in 52% of cases. Risk factors included hormonal contraceptive use, family history, and cyclosporine therapy. Ultrasonography was the most common diagnostic tool, followed by MRI and FNAC. Treatment was mainly surgical, with recurrence noted in 15.4% of patients. There was no evidence to suggest that the risk of cancer in MFA was greater than that of solitary FA. The review highlights inconsistencies in MFA definitions and calls for consensus on diagnostic criteria, prospective research, and the creation of standardized treatment algorithms to improve care and outcomes for this particularly distressing subset of benign breast diseases.

## 1. Introduction

Fibroadenomas are common, benign breast lesions regarded as aberrations of normal growth and involution of the breast [1]. Although fibroadenomas most commonly occur in the third decade of life, they also occur in adolescence, accounting for 44%–94% of all breast biopsies in this age group compared to 30%–50% of all breast biopsies in general [2]. Adolescents exhibit unique manifestations of fibroadenomas, contributing to the use of confusing and complex terminology in literature. While a typical simple fibroadenoma presents as a solitary, well‐encapsulated breast lump measuring 2–3 cm, several variants exist in this age group. Juvenile fibroadenomas are rapidly growing lesions [3], often associated with skin changes like stretching, shininess, dilated veins, and inflammatory signs. Giant fibroadenoma is a fibroadenoma over 500 g, greater than 5 cm in size, or replacing at least four‐fifths of the breast; when associated with skin changes in adolescents, it is called a “giant juvenile fibroadenoma” [4].

Additionally, multiple fibroadenomas (MFAs) are found in 10%–15% of patients and may present in the same or both breasts [5, 6]. Unlike the other variants mentioned above, there is considerable heterogeneity in how MFAs are defined across studies, leading to ambiguity regarding what constitutes MFA. For example, in a retrospective analysis of 80 adolescents with fibroadenomas, 10% had bilateral lumps, and 2.5% had MFA [7], while in other studies even bilateral FA may be considered MFA. In contrast to solitary fibroadenomas, which have a well‐defined natural history, management strategy, and prognosis [8], MFAs remain poorly characterized and lack an established protocol. The absence of uniform definitions and outcome‐based data represents a critical research gap, limiting evidence‐based guidelines regarding surveillance and interventions with regards to MFA. Therefore, this study aimed to conduct a systematic review of literature on MFA, with the primary objective being to standardize terminology that defines MFA and highlight clinical implications for management.

## 2. Methods and Materials

### 2.1. Literature Search Strategy

An electronic search of PubMed (NLM), Medline (Ovid; Wolters Kluwer), Scopus (Elsevier), and Embase (Elsevier) databases was conducted according to the PRISMA statement, 2020 [9]. An initial search using the MeSH (medical subject headings) term “multiple fibroadenoma∗”[tiab] AND breast[tiab] yielded 46 articles. As this condition is not well defined, we expanded our search to include “multiple fibroadenoma^∗^ AND breast,” yielding 419 articles. Filters were applied to limit the search to English‐language publications, studies on human subjects, and the period from January 1948 to June 2025, resulting in 312 articles. The search was conducted independently by two study authors (Prathiksha Math and Gayathri Sajith). All titles and abstracts were thoroughly reviewed, and appropriate studies were assessed for inclusion. The reference sections of all studies were also assessed to ensure the identification of relevant studies. In cases of differences between the first and second authors regarding the inclusion or exclusion of studies, a third author (Kiran Mahadevappa) reviewed the articles to adjudicate and provide a final decision, ensuring consistency and accuracy in the review process.

### 2.2. Inclusion and Exclusion Criteria

**FIGURE 1:**
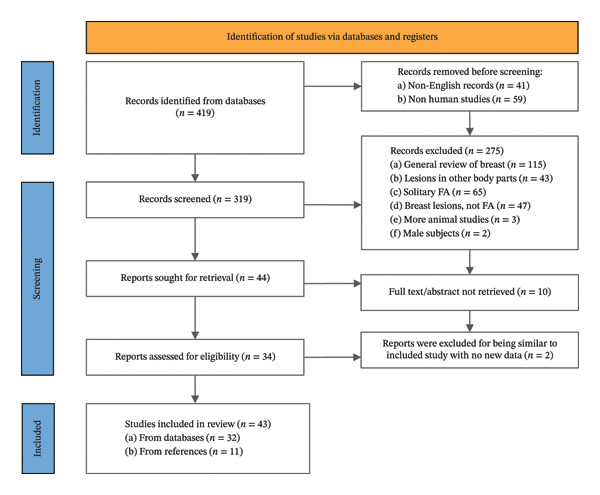
Preferred Reporting Items for Systematic Reviews and Meta‐Analyses (PRISMA) flow diagram.

Studies were included if they described MFA in the breast. Studies describing other breast diseases with only a passing reference to fibroadenoma, describing solitary fibroadenoma, or describing diseases not primarily in the breast were excluded. The characteristics of the excluded studies are shown in Figure 1.

Data were systematically extracted after the examination of individual manuscripts. Study details and patient details, including age, race, laterality, number, associated features, and risk factors, if any, were recorded. Multiple investigative modalities and surgical techniques used for the management of MFA, along with the observed complications, were noted. Recurrence rates and median times to recurrence were recorded when data were available.

### 2.3. Quality Assessment

The heterogeneity of the included studies prevented a formal risk of bias assessment. However, the level of evidence was independently assessed by two reviewers using the Oxford Centre for Evidence‐Based Medicine [10] (Table 1).

**TABLE 1:** Study characteristics.

Study	Study design	No. of patients	Level of evidence (OCEBM)
Amshel et al. [5]	Case report	1	Level 5
Muttarak et al. [6]	Case report	1	Level 5
Wiegenstein et al. [7]	Case series	12	Level 4
Morrisand Kelly [8]	Case report	2	Level 5
Im et al. [11]	Case report	1	Level 5
Williamson et al. [12]	Case report	1	Level 5
Mashoori et al. [13]	Case report	1	Level 5
Grouthier et al. [14]	Cohort	72	Level 3
Kobayashi et al. [15]	Case report	1	Level 4
Brahmachari et al. [16]	Prospective cohort	30	Level 3
Lee et al. [17]	Case report	1	Level 4
Kaur et al. [18]	Case report	1	Level 4
Panda et al. [19]	Case report	1	Level 5
Chakhtoura et al. [20]	Multicentric cross‐sectional study	71	Level 2
Povoski [21]	Case report	1	Level 4
Schneider et al. [22]	Case report	1	Level 4
Traoré et al. [23]	Case report	1	Level 5
Calabrese [24]	Cross‐sectional study	17	Level 3
Kuijper [25]	Case report	1	Level 5
Jain et al. [26]	Case report	1	Level 5
Ismail et al. [27]	Case report	1	Level 5
Wang et al. [28]	Nonrandomized controlled trial	62	Level 3
Rai et al. [29]	Randomized control trial with triple blinding	38	Level 2
Darwish et al. [30]	Case report	1	Level 5
Stefanon et al. [31]	Case report	1	Level 5
Camara et al. [32]	Case series	2	Level 4
Shibata et al. [33]	Case report	1	Level 5
Cyrlak et al. [34]	Case report	1	Level 4
Baildam et al. [35]	Prospective cohort	10	Level 2
Rao et al. [36]	Case report	1	Level 5
Zhang et al. [37]	Case report	1	Level 5
Baxi et al. [38]	Case report	1	Level 5
Yue et al. [39]	Prospective cohort	20	Level 3
Naraynsingh and Raju [40]	Case series	3	Level 4
Lai et al. [41]	Retrospective cohort	20	Level 3
Zhao et al. [42]	Case report	1	Level 5
Agodirin et al. [43]	Case series	3	Level 4
Singh et al. [44]	Case report	1	Level 5
Lu et al. [45]	Retrospective cohort	39	Level 3
Li et al. [46]	Prospective cohort	65	Level 3
Cant et al. [47]	Prospective cohort	43	Level 3
Nyati et al. [48]	Case report	1	Level 5
Silfen et al. [49]	Case report	1	Level 5

### 2.4. Statistical Analysis

Data on individual patients were tabulated. Categorical variables were presented as frequencies and percentages, and continuous variables as mean ± standard deviation. A descriptive analysis was performed to determine the frequencies of age at presentation, number of fibroadenomas, side of the lesion, size of the lesion, recurrence, use of oral contraceptive pills (OCPs), investigations, and interventions. When data were available only for a subset of the total patients in a study, only those with MFA were included in the analysis. Multivariate analysis was not possible because most data sources were case reports or case series. Statistical significance was set at *p* < 0.05. Data were analyzed using STATA software (StataCorp; 2019, *Stata Statistical Software: Release 16*. College Station, TX: StataCorp LLC).

## 3. Results

Initial search results revealed a total of 412 manuscripts. Of these, 43 manuscripts were ultimately eligible for inclusion in the systematic review (Figure 1). The majority were case reports (*n* = 27, 62.8%), followed by cohort studies (*n* = 8, 18.6%), case series (*n* = 4, 9.3%), cross‐sectional studies (*n* = 2, 4.7%), and interventional trials (*n* = 2, 4.7%).

### 3.1. Epidemiology and Clinical Presentation

A total of 48 unique patients from 31 case reports and series (mean age 22.79 ± 9.30 years) were included that described the demographic and clinical characteristics of patients with MFA (Table 2). Adolescents were most commonly affected (54%), with the earliest presentation being at 11 years of age. Approximately half of the reported adolescent cases presented with > 10 lesions (*n* = 25, 52.08%), with a significant percentage having bilateral disease (*n* = 31, 64.58%). The largest number of discrete lesions reported was 50, although other articles mentioned “innumerable lesions” interspersed with minimal normal breast tissue.

**TABLE 2:** Demographic and clinical characteristics of 48 unique patients included from case reports and case series.

Variable	*n* (% of total)
Age distribution (years)	
11–20	26 (54.2)
21–30	12 (25.0)
31–40	7 (14.6)
> 40	3 (6.3)
Side	
Bilateral	31 (64.6)
Unilateral (NS)	11 (22.9)
Right	4 (8.3)
Left	2 (4.2)
Number of lesions	
< 3	7 (14.6)
3–5	10 (20.8)
6–10	4 (8.3)
> 10	25 (52.1)
NS	2 (4.2)
Diameter of largest lesion	
< 2 cm	0 (0.0)
2–5 cm	16 (33.3)
5–8 cm	4 (8.3)
8–10 cm	4 (8.3)
> 10 cm	4 (8.3)
Axillary tail involvement	7 (14.6)
Recurrence	5 (10.4)^∗^
Risk factors	
Hormonal pills for contraception or infertility	14 (29.2)
Family history	7 (14.6)
Cyclosporine	4 (8.3)
Large breasts	3 (6.3)
Black race	2 (4.2)
Pregnancy	2 (4.2)
Syndromes	2 (4.2)
Early menarche	1 (2.1)
NS	13 (27.1)
Associated conditions	
Idiopathic hemihypertrophy	1 (2.1)
HIV	1 (2.1)

*Note: n*, no. of patients.

^∗^Recurrence rate derived from only case reports and case series.

### 3.2. Imaging Findings

The majority of studies reported the use of ultrasound (US) for lesion assessment (no. of patients = 457, pooled percentage = 86.5%). Other common imaging modalities used were MRI breast and mammography (Table 3).

**TABLE 3:** Preoperative investigations performed across manuscripts included in the review.

Investigation	No. of studies	Indication	Studies excluded	No. of patients (*N* = 528)	Pooled percentage (%)
US	28	Routine imaging	0	457	86.5
MRI	9	Assess radiological features	0	122	23.1
Core biopsy/FNAC/incision biopsy	20	Preoperative histopathology	0	115	21.7
Hormonal assay	4	H/O infertility treatment and early menarche, academic	1^∗^	83	15.7
Genetic testing and chromosomal analysis	3	Unusual presentations like idiopathic hemihypertrophy, innumerable lesions in a young patient, academic	0	73	13.8
Mammography	10	Screening: to rule out malignancy by imaging	0	36	6.8
IHC	9	Coexisting breast cancer, academic	4^∗∗^	35	6.6
Electron microscopy	2	Academic	0	22	4.1
Doppler	3	As part of ultrasound ablation	0	22	4.1
Cytogenetic analysis of specimen	1	Academic	0	17	3.2
CT	1	Metastasis workup	0	1	0.1
Clonality analysis	1	Academic	0	1	0.1
Not mentioned	2	—	0	44	8.3
Immunofluorescence	1	Academic	1^†^	0	0

*Note: N*, total number of patients.

Abbreviations: CT, computerized tomography; FNAC, fine needle aspiration cytology; IHC, immunohistochemistry; MRI, magnetic resonance imaging; US, ultrasound.

^∗^Hormone name not mentioned.

^∗∗^ER/PR status not done/mentioned.

^†^Not done in human subject.

### 3.3. Management

Surgical intervention was reported in 79% of manuscripts (*n* = 34) and conservative management in 23.2% (*n* = 11), while 11.62% (*n* = 3) of the manuscripts did not address management. Eleven studies delved into specific treatment options and subsequent complications (Table 4).

**TABLE 4:** Treatment modalities used across manuscripts included in the review.

Treatment	No. of studies	No. of patients
Surgical		
Limited excisions		
Excision	15	70
Round block technique excision	1	20
CAST	1	3
Extensive excisions		
Mastectomy	8	8
Ribeiro Razai	1	2
Excision en bloc	1	1
Central pedicle reduction mammoplasty	1	1
B/L gland resection and mammoplasty	1	1
Minimally invasive techniques		
RFA	1	65
UGVAE	1	62
Endoscopic resection via gasless transaxillary approach	1	39
FUAS	1	20
VAM	1	1
Conservative		
Ormeloxifene	2	68
Progestin	1	72
Tamoxifen (adjuvant)	1	1
Bromocriptine (adjuvant)	1	1
Observation	3	7
Discontinuation of cyclosporine	3	3
Not specified	3	82

Abbreviations: CAST, circumareolar incision‐subdermal tunneling dissection; FUAS, focused ultrasound ablation surgery; RFA, radiofrequency ablation; UGVAE, USG‐guided vacuum‐assisted excision; VAM, vacuum‐assisted mammotome.

## 4. Discussion

This systematic review on MFA demonstrates that the current evidence base is largely limited to Level 4 or 5 studies. While this allows a preliminary overview of demographic features and clinical presentation, the predominance of low‐quality evidence limits confidence in broader inferences. Important gaps identified were a standard definition of the condition “multiple fibroadenoma,” differences in pathogenesis of MFA versus solitary fibroadenoma, management strategy, recurrence rate, and associated breast cancer risk. In the articles selected for this review, the term “multiple fibroadenoma” was inconsistently defined by different authors. While some used it to describe two to five fibroadenomas [7, 11–13], it was uncertain whether this number was with regard to a single breast or to the individual. In other studies, the term MFA was used for the presence of at least 3 fibroadenomas in one breast [14–16], and it was still unclear whether the 3 lesions were present at the same time or appeared over a period of time. This confusion is compounded by the grammatical ambiguity of the term “multiple.” Such heterogeneity in definitions has important clinical and research implications. In the absence of standardized terminology, comparisons across studies become unreliable, pooled analysis is limited, and meaningful conclusions regarding natural history, recurrence risk, risk for development of cancer, surveillance strategies, and indications for interventions cannot be made. From a clinical standpoint, inconsistent definitions may lead to variability in counseling, treatment decisions, and follow‐up protocols, potentially resulting in both inadequate surveillance and overtreatment. Recognizing MFA as a distinct clinical entity with unique implications for management, therefore, necessitates a clear, reproducible, and clinically relevant nomenclature. We propose the following:

 Fibroadenoma: a single fibroadenoma in one breast.
 Unilateral fibroadenomas: two fibroadenomas in a single breast.
 Bilateral fibroadenomas: two fibroadenomas, one in each breast.
 MFAs: three or more fibroadenomas in one or both breasts, either diagnosed simultaneously or over a period of time. This definition would encompass
  (a) At least 3 fibroadenomas in a single breast, irrespective of their temporal presentation.
  (b) Two in one breast and at least one in the contralateral breast, meeting the numerical threshold across both breasts.
  (c) Two fibroadenomas, one in each breast, with a history of surgical excision to remove a similar lesion in either breast.
  (d) A single fibroadenoma with a history of surgical excision of two or more histologically similar lesions removed concurrently or at different time points.

### 4.1. Theories of Pathogenesis and Risk Factors

Fibroadenoma is regarded as an aberration of normal development and involution, but this pathogenesis appears to be exaggerated in MFA by a field effect, characterized by a heightened sensitivity of the breast tissue to hormonal fluctuations. Like solitary fibroadenoma, MFAs are also seen only in the reproductive age group. There does not appear to be a correlation with the duration of hormonal exposure, as MFA can be seen as early as within a year of attaining menarche [17]. Hormonal theories suggested include high estrogen and progesterone receptor expression [12], hypersensitivity of the breast to estrogen [18, 19], disproportionately high estrogen levels in the local breast tissue, increased prolactin (PRL) levels, and expression of PRL receptor gene variants that may induce tumorigenesis [20].

High ER/PR expression supports the hypothesis that hormonal stimulation may play a role in the development and growth of MFA, aligning with studies suggesting a potential association between OCP use and MFA risk [7, 12, 16, 21, 22]. However, given the low level of evidence of these studies, further research is needed in the form of a well‐designed case–control study to establish an odds ratio between OCP use and the development of MFA. This is true even for the use of clomiphene, which was identified as a risk factor in a case report [15]. The theory of hypersensitivity of the breast tissue to estrogen or that of local hyperestrogenism in the breast gains leverage with the beneficial effects of antiestrogens or progestins, which are thought to restore the hormonal balance, as well as in reports regarding the use of cyclosporine in renal transplant patients (see medications below). The incidence of developing MFA was 4.6% in women who were on cyclosporin following organ transplantation over a mean follow‐up period of 4 ± 1.2 years [50].

Despite strong experimental evidence of PRL‐induced mammary tumorigenesis [51], the average circulating levels of PRL in two groups of women, one with MFA and a control group, were not significantly different (12.7 ± 13.0 ng/mL vs. 14.4 ± 9.2 ng/mL, respectively). In addition, although prolactin receptor (PRLR) gene variants PRLR_I146L_ and PRLR_I176V_ were previously identified in a cohort of 95 women with MFA, a study comparing women with MFA to control women did not find a higher prevalence of these PRLR variants in the MFA group. In the same study, transgenic mice were generated that expressed the PRLR_I146L_ variant, but no evidence of tumorigenesis was found [20]. A single case report by Traoré et al. describes a pregnant woman with gigantomastia and MFA associated with hyperprolactinemia [23]. However, the PRL levels reported fall within the normal physiological range for the third trimester of pregnancy.

### 4.2. Pathology, Recurrence, and Risk of Cancer

A cytogenetic study of excised MFA (*n* = 3) in a single subject revealed an identical translocation (t11; 12) (q21; q15) in all specimens, suggesting a clonal origin of MFA [24]. However, MFA can be associated with other types of breast pathologies, particularly phyllodes tumors, which have been reported in 6.3% of cases [14]. A benign phyllodes tumor can occur concurrently with MFA or present subsequently in the same woman [15]. Additionally, malignant phyllodes tumors, carcinoma in situ, and invasive breast cancer have been concurrently diagnosed in women with MFA [25–27]. The breast stroma and epithelium may undergo separate molecular changes during neoplastic processes, leading to multiple breast lumps bearing distinct pathologies. Therefore, all excised breast lesions in the setting of MFA should undergo comprehensive histopathological evaluation, particularly in women with high‐risk factors, breast cancer, or prior hormonal therapy exposure. Given the possibility of coexisting malignancies, complete tissue sampling of all excised masses is essential, as routine sectioning may be insufficient for accurate carcinoma detection.

Postoperative recurrence of MFA can either be a true recurrence due to incomplete excision or a new lesion due to the multicentric nature of the disease. Incomplete excision was described by Wang et al. [28] during the use of a minimally invasive technique (vacuum excision, see below), where 5 out of 60 subjects were observed to have impalpable residual lesions on US (11.2 ± 3.0 mm, range 8–16 mm) after 12 months of excision. Five other studies in the review discussed recurrence in the same breast, but it was unclear whether these were true recurrences at the original surgical site or new lesions arising elsewhere in the same breast. Recurrence in these studies was noted at a median (IQR) of 8.5 (5.25, 38.75) months from the first surgery. Four of the five studies reported excision of recurrent lesions measuring 2–15 cm. Only one case report [12] mentions conservative management, where the patient developed 19 new but small lesions (size ranging from 1 to 1.5 cm) over a period of 10 years. Notably, an increase in local recurrences has been associated with a higher risk of malignant transformation, underscoring the importance of long‐term surveillance in affected individuals. In a case report of a 17‐year‐old girl operated on for the fourth time with MFA, a cystosarcoma phyllodes was diagnosed [27]. The recurrence rate calculated as a pooled percentage from these 6 articles (*n* = 10, total number of patients = 65) was 15.4%, which, due to the lack of long‐term studies, may not reflect the true recurrence rate.

Malignant transformation in a solitary fibroadenoma is rare, with an estimated incidence of less than 0.3% [18]. However, the risk is higher in women older than 40 years, with a reported incidence of 1.7% [19, 52]. MFAs are more likely to exhibit complex histopathological features such as cystic changes, sclerosing adenosis, epithelial calcifications, or papillary apocrine changes, which are risk factors for malignant transformation [18]. We found no evidence to suggest that MFAs have a higher rate of malignant transformation than solitary FAs.

### 4.3. Medications—Ormeloxifene, Tamoxifen, Withdrawal of Cyclosporine

In a prospective cohort study of 30 women with MFA, when ormeloxifene, a selective nonsteroidal nonhormonal antiestrogen (selective estrogen receptor modulator), was administered at 30 mg every alternate day for 3 months, it reportedly induced complete dissolution or > 50% size reduction of lesions on US at the end of 6 months in 34% of the women. Partial response, defined as a < 50% size reduction, was seen in 46% of women, and no change was observed in 17%. Only 1 patient had an increase in the size of the lesion. No side effects were reported. The limitations of this study include the absence of a control group, potential inter‐ and intra‐individual variability in US measurements, and the absence of follow‐up data to assess long‐term outcomes and disease progression. A recently published triple‐blinded randomized controlled trial with 104 subjects also concluded that ormeloxifene (in the same dose as above) significantly reduced size, mastalgia, and psychological distress compared to a placebo [29]. The mean age of the women was 29.9 ± 9.65 years, and 73.1% had MFA at recruitment. The volume of the fibroadenoma by US in the intervention group reduced from 3.67 ± 1.65 cm^3^ at baseline to 1.92 ± 1.04 cm^3^ after 12 weeks of follow‐up (*p* = 0.042), compared to 3.12 ± 1.16 cm^3^ to 2.73 ± 0.78 cm^3^ (*p* = 0.781). The number of patients who achieved > 50% reduction in volume in the intervention group was also significantly higher than that in the control group (28.8% vs. 13.5%, *p* = 0.007). The limitations of this study include the absence of follow‐up after drug cessation and intraindividual variability in US measurements. Tamoxifen, another drug of the same class, was used in a case report of a 16‐year‐old girl with approximately 30 MFA. Larger MFAs (1–11 cm) were excised, and she received postoperative Tamoxifen at a dosage of 20 mg/day for 2 years, following which it was noted that small MFAs (< 1 cm) had resolved and no new lesions were detected on US. The only side effect observed was mild hot flushes. No follow‐up was reported after stopping the drug. Progestins are another drug class that caused a decrease in the size of MFA on US at −0.1 ± 0.02 mm per month over a 10‐year‐period (*p* < 0.0001), although there was no change in the number of lesions. In this study, however, each of the 65 women included could have received different types of treatment with respect to the dose, type, and duration of progestins. There were no data available regarding the total duration of exposure to progestins or side effects.

In a prospective cohort of 39 women under 55 years of age who had undergone renal transplantation, 29 were either actively receiving cyclosporine or had been on this medication within the last 6 months. Among these, 45% were diagnosed with MFA, with a mean post‐transplant duration of 57.8 ± 33.4 months at detection. Comparison between women who developed MFA and those who did not revealed significantly lower FSH values in the MFA group (4.7 ± 2.3 vs. 22.2 ± 21.9 mU/L, *p* < 0.01) with a state of hyperestrogenism (serum estradiol 569 ± 162 vs. 283 ± 61 pmol/L, *p* < 0.05). The withdrawal of this drug in women who developed MFA after renal transplantation was considered in three studies [6, 30, 31]. While cessation of the drug prevented the development of new lesions at 6 months’ follow‐up, it did not lead to regression of existing ones. Further research is warranted to elucidate the potential hormonal mechanisms underlying cyclosporine‐induced MFA development.

Unlike fibrocystic disease, there is no clinical indication for the use of vitamin E or evening primrose oil in the management of MFA.

### 4.4. Investigations

As with any breast lesion, imaging is essential as a part of the triple assessment. In young individuals, US remains the first‐line modality owing to its easy accessibility, minimal radiation exposure, and cost efficiency. MRI of the breast offers enhanced visualization of complex lesions, aiding the choice of an optimal surgical approach [32], but does not appear to offer any additional information for routine management. It has been typically used when the breast appears to be studded or completely replaced with MFA [11, 22, 33] or when the average number of lesions in a single breast is ≥ 10 [20, 32]. Although MRI can detect lesions as small as 2–5 mm [14, 22, 33], it cannot differentiate between malignant and vascular benign lesions and tends to show a vaguely defined area of enhancement instead of discrete lesions when the MFAs are closely approximated. Mammography is not routinely recommended for MFA but has been done as part of regular screening for women over 40 years old [53] and in cases of suspected malignancy, cyclosporine therapy [34], family history of cancer, or atypical US findings like calcifications.

Hormonal assays (FSH, LH, estradiol, and PRL) are not routinely done for MFA. When done, usually in the background of other hormonal complaints like ovarian hyperstimulation syndrome (OHSS) and polycystic ovary syndrome (PCOS), they have been observed to be normal. Only one study conducted on women who developed MFA or FA while on cyclosporine therapy (*n* = 29) reported significant hormonal changes compared to women on cyclosporine who did not develop MFA (*n* = 16) [35]. The MFA group showed a lower average serum FSH level and a higher average serum estradiol compared to the non‐MFA group (4.7 ± 2.3 mU/L vs. 22.2 ± 21.9 mU/L; *p* < 0.01 and 569 ± 162 pmol/L vs. 283 ± 61 pmol/L; *p* < 0.05, respectively). These findings point out a hormonal basis for cyclosporine‐induced MFA development. PRL has been discussed under pathogenesis (see above). These findings suggest that there is currently no evidence to support measuring hormonal levels as part of clinical evaluation of patients with MFA, except in those on cyclosporin or those with concomitant conditions like PCOS or OHSS.

IHC to assess ER/PR status is recommended in patients with a family history of cancer, recurrent MFA, or MFA concurrent with breast cancer or phyllodes tumors [15, 20, 25, 27, 36, 37], as this may inform postoperative treatment strategy. Among the studies under this systematic review, Level 3 evidence to determine ER/PR status for all MFA comes from a cohort study, which showed that ER/PR positivity of the excised tumors was 85% and 98%, respectively [14]. It represents the highest level of evidence among the existing literature, as the remaining studies consist of four case reports (with a minimum lesion number of 5) with variable findings. ER and PR were positive in one study [37], ER/PR was negative in another [38], ER was negative and PR was positive in the third [12], and there was only reporting of ER positivity in the fourth [15].

This literature review found no justification for routine genetic testing in MFA. Only a single case report identified a PTEN mutation in an 18‐year‐old girl with a family history of cancer unrelated to Cowden syndrome. The article notes that although genetic testing was performed despite the family history not meeting the guidelines of the National Comprehensive Cancer Network, PTEN mutations can be associated with benign breast disease in 35% of the carriers [11]. Doppler and CT are not routinely recommended and are reserved for the detailed evaluation of tumoral vessels when planning USG‐guided therapy and the evaluation of metastasis in concurrent cancer, respectively [22, 27, 37, 39].

### 4.5. Surgery

Various surgical strategies have been explored for MFA to alleviate symptoms such as disfigurement, pain, risk of concurrent cancer, and psychological distress, particularly in juvenile MFA cases. Besides standard subcutaneous mastectomy and en bloc excisions, other techniques have been described for MFA. Rezai’s technique, involving preoperative freehand marking and modulating the inferior pedicle, is noted for its safety. Other methods include circumareolar incision [40], round block technique [41], reduction mammoplasty and mastopexy [42], and the CAST method [43]. However, these surgical interventions may not be ideal for women with smaller breasts [21] or in cases of recurrent MFA. Complications such as ductal damage, hypertrophic scarring [18], nipple necrosis, ecchymosis [41], edema, bruising [43], and lymphangitis [44] also highlight the need for alternative therapeutic approaches. In patients with a tendency for recurrence, surveillance can be recommended as for solitary FA. A volume growth rate of less than 16% per month in those younger than 50 years and less than 13% per month in those 50 years or older on US is an acceptable long‐term rate of growth. Practically, during follow‐up, a change in size of ≤ 0.2 cm in all dimensions or a growth rate of ≤ 20% for a 6‐month interval for all ages is acceptable and does not raise a red flag for a repeat biopsy [45].

### 4.6. Minimally Invasive Techniques

In adolescents with MFA in particular, endoscopic resection via a transaxillary approach (gasless) has shown a significantly lower complication rate of 11.1% compared to 66.6% (*p* = 0.011) of traditional open surgery, in addition to shorter operative times and smaller incisions [54]. Newer minimally invasive procedures have also been investigated as treatments for MFA, which are performed under local anesthesia, have improved cosmetic outcomes but may require multiple sessions. Ultrasound‐guided vacuum‐assisted excision (UGVAE) for MFA was first described in a case report of a single patient [21], where it was used to successfully excise 14 lumps in 7 sessions over a period of 10 months. Both breasts were involved, and the average size of the lesions was 1.1 ± 0.5 cm. The largest lesion measured 2.3 cm and required 32 cores (8 gauges) to be harvested for complete excision. A follow‐up US performed 8 months after the last session revealed no recurrences or new lesions. Level 3 evidence for UGVAE comes from a prospective nonrandomized study [28] that compared this technique with conventional open excision (62 patients with 150 lesions vs. 36 patients with 87 lesions). The two groups did not have any significant differences in the age of the subjects, number, and size of lesions. Although this study compared women with multiple benign breast lumps, fibroadenomas were the most common pathology (68%). The comparison showed a slightly higher incomplete removal rate at 6 and 12 months on US in UGVAE compared to conventional open excision, which was not statistically significant (8.3% vs. 0%, *p* = 0.16). As the average size of the residual lesions was 25.2 ± 3.7 mm (range 21–30 mm), the authors concluded that the optimal candidates for complete resection by UGVAE are patients with lesion sizes < 20 mm. Complications in the UGVAE group included procedural bleeding (4.8%), postprocedure ecchymosis (6.5%), and ecchymoma (1.6%), which were all self‐limiting and required no further intervention other than compression in the immediate postprocedure period. Ablative procedures may offer superior cosmesis compared to vacuum excision owing to the use of a fine 19G puncture electrode. The authors claim that skin integrity and shape are better maintained, and there are no complications like bleeding and inadvertent damage to mammary ducts. The procedure of RFA in MFA was evaluated in a cross‐sectional study of 65 women with 256 MFA with a mean lesion size of 11.68 ± 6.26 mm [46]. All women underwent US‐guided placement of the RFA electrode at the distal end of the FA capsule under GA, followed by application of energy at 10–15 W with a center temperature of up to 65°C–95°C. The duration of treatment per lesion in unit time was not mentioned in the study but was said to be continued until no perfusion was detected on contrast‐enhanced US. Postprocedure, all women received local cold packs for 6 h and the US estimation of volume at 1 and 3 months. The volume reduction rate improved to 75.9% at 3 months compared to 39.06% at 3 months, with complete absorption observed in 17.58% of the lesions. In 3 subjects mild skin hyperemia was observed, which resolved spontaneously in a week. Complications such as skin burns, hematoma, or nipple discharge (secondary to RFA performed close to the areola) were not observed in any of the subjects.

Focused ultrasound ablation surgery (FUAS) has also been recently used to manage MFA in 20 women in China, where the median size of the lesion was 14.0 (11.0, 20.1) mm [39]. The subjects were placed in a prone position, and the US waves were focused on the deep side of the target FA while traversing through a low‐temperature degassed water medium, requiring a median treatment time of 13.0 (6.0, 23.0) min per lesion. The median shrinkage rate was 66.4 (43.6, 89.5)% in the 12 months of follow‐up on the US. Besides mild pain noted in 72.3% of the subjects, no other complications were noted.

### 4.7. Limitations and Future Directions

The current reliance on case reports and case series limits generalizability, introduces selection and reporting bias, and precludes robust assessment of causality, recurrence patterns, and treatment effectiveness. Considering the predominance of low‐level evidence, the present work synthesizes the available literature and highlights substantial evidence gaps, underscoring the need for higher‐quality studies before firm clinical recommendations can be made. Future research should prioritize the adoption of standardized diagnostic and reporting criteria and prospective multicenter registries to better characterize the natural history, recurrence risk, and long‐term outcomes of MFA.

## 5. Conclusion

MFA represents a distinct clinical entity within benign breast disease, affecting adolescents and young women during hormonally dynamic phases of life. This review provides a structured synthesis of existing literature, underscoring the lack of consensus on the definition and diagnostic criteria for MFA, as well as the need for a standardized imaging and follow‐up protocol. Although the risk of malignancy in MFA remains low, psychological distress, cosmetic concerns, and surgical implications necessitate a nuanced, individualized approach. By highlighting substantial evidence gaps, this review also emphasizes the need for higher quality, prospective studies to establish evidence‐based guidelines for evaluation and long‐term care.

## Author Contributions

Prathiksha Math and Shalini G. Hegde conceptualized the study, Prathiksha Math and Gayathri Sajith conducted the systematic review, Prathiksha Math wrote the first draft, and Gayathri Sajith contributed to formatting and revising the manuscript. Kiran Mahadevappa and Shalini G. Hegde supervised the conduct of the review and contributed to critically reviewing and revising the manuscript.

## Funding

No funding was received for this manuscript.

## Disclosure

All authors approved the final manuscript as submitted and agreed to be accountable for all aspects of the work.

## Conflicts of Interest

The authors declare no conflicts of interest.

## Data Availability

The data used in this study are available on reasonable request to the corresponding author.
